# Electrochemical migration behavior of SnAgCuNi solder alloy in a simulated dew condensation environment

**DOI:** 10.1039/d5ra06195d

**Published:** 2025-10-13

**Authors:** Luntao Wang, Xuan Liu, Ziheng Zhao, Jialiang Song, Yuxin Shu, Honglun Wang, Yao Tan, Hao Zhang, Heqian Wang, Junsheng Wu, Kui Xiao

**Affiliations:** a Institute for Advanced Materials and Technology, University of Science and Technology Beijing Beijing 100083 China xiaokui@ustb.edu.cn; b XiaMen Golden Egret Special Alloy Co., Ltd XiaMen 361007 China; c Xichang Satellite Launch Center Xichang 615000 China; d China National Electric Apparatus Research Institute Co., Ltd, National Key Laboratory of Environmental Adaptability for Industrial Products Guangzhou 510663 China

## Abstract

This study investigates SnAgCuNi lead-free solder alloys, focusing on the electrochemical migration (ECM) failure mechanism under simulated dew condensation conditions. The corrosion characteristics and ECM resistance of the alloys were systematically evaluated. Electrochemical measurements showed that low Ni additions (0.05–0.10 wt%) shifted the corrosion potential positively, reduced the corrosion current density, and significantly increased the charge transfer resistance, indicating improved passivation behavior. The results indicate that the addition of 0.05–0.10 wt% Ni effectively suppresses the ECM reaction rate, reduces ion concentration within the droplet system, mitigates corrosion of the solder alloy, and consequently enhances the ECM resistance of SAC305. In contrast, increasing the Ni content to 0.25 wt% markedly deteriorates ECM resistance. These findings provide a scientific basis and preventive strategies for addressing potential reliability concerns and the risks of metal corrosion migration in electronic packaging.

## Abstract 2

This study investigates SnAgCuNi lead-free solder alloys, focusing on the electrochemical migration (ECM) failure mechanism under simulated dew condensation conditions.

## Graphical abstract

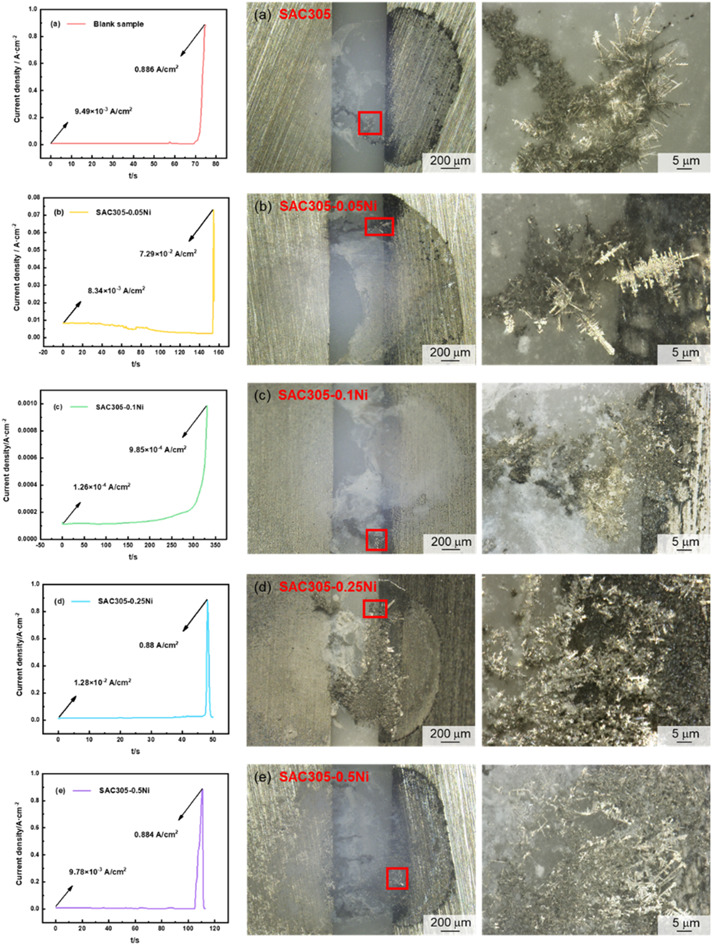

## 1 Introduction

Soldering is a critical process for component packaging and interconnection in electronic devices, and the manufacturing yield and service life of microelectronic devices largely depend on the reliability of miniature solder joints. Solder alloys not only provide electrical interconnections but also provide essential mechanical support. With the enforcement of regulations restricting the use of lead-containing interconnection materials, the demand for lead-free alternatives in consumer electronics has risen sharply. To meet this demand, a variety of lead-free solder systems have been developed to replace conventional Pb–Sn solders, including Sn–Cu, Sn–Ag, Sn–Zn, Sn–Bi and Sn–Ag–Cu alloys.^1–5^ Among these, the ternary Sn–Ag–Cu (SAC) alloy has gained prominence due to its relatively low melting point, high bonding strength, excellent electrical conductivity, and moderate cost, making it one of the most widely applied and extensively studied lead-free solders.^6–8^ Nevertheless, its microstructure tends to form needle-like Ag_3_Sn and coarse Cu_6_Sn_5_ intermetallic compounds, which can significantly degrade mechanical performance.^9,10^ Minor alloying strategies have been employed to refine microstructure and enhance performance. In particular, trace Ni additions to SAC305 solder improve tensile strength, extend service life, and enhance corrosion resistance and solderability.^11–13^

In parallel with mechanical and thermal considerations, the rapid advancement of electronic systems has driven components toward miniaturization and higher integration density, placing greater reliability demands on solder joints. Electrochemical migration (ECM) is one of the corrosion failure modes to which Sn-based solder alloys are most susceptible. On one hand, certain electronic devices operate for extended periods in alternating hot–humid environments, where condensation can occur and exacerbate failure risks. On the other hand, when conductive contaminants are present on the solder surface, ion migration may take place under an applied potential difference, leading to poor joint bonding or corrosion-induced short circuits. To date, most reliability studies on multicomponent solder alloys have focused on thermal cycling and mechanical endurance,^14,15^ whereas investigations into ECM resistance under condensation environments remain limited.

Previous studies have demonstrated that ECM behavior strongly depends on solder alloy composition, with different alloying elements exhibiting distinct dissolution and migration characteristics. In Sn–Pb alloys, Pb is the primary migrating element in pure water, and its migration sensitivity is higher than that of Sn in eutectic compositions.^16,17^ However, in sulfate-containing solutions, the formation of insoluble PbSO_4_ or PbO layers protects Pb from dissolution, in contrast to the behavior observed in chloride solutions and pure water.^18,19^ For Sn–Ag alloys, the presence of a stable Ag_3_Sn intermetallic compound suppresses Ag migration, and Ag additions extend the time-to-failure of Sn–3.5Ag and Sn–3Ag–0.5Cu alloys in 0.001 wt% Na_2_SO_4_ solution, with the degree of migration resistance closely related to Sn dissolution characteristics.^20,21^ In Sn–Zn alloys, Sn–9Zn exhibits properties similar to eutectic Sn–Pb solder, and the addition of Zn to Sn–Bi-based solders significantly reduces the time-to-failure under high-temperature and high-humidity conditions, thereby lowering ECM resistance.^22^ However, in Sn–9Zn alloys, Sn remains the dominant migrating element because the Zn-containing passivation film suppresses Zn dissolution. Given these findings, further investigation into the ECM behavior of Ni-modified SAC alloys under condensation conditions is warranted, as it may provide insights into optimizing solder composition for enhanced corrosion resistance and long-term reliability in humid service environments.

In this study, an *in situ* observable water drop test was employed to simulate the ECM failure behavior of SnAgCuNi solder alloys under dew condensation conditions. The corrosion resistance of the alloys was first assessed by electrochemical measurements. A stereomicroscope was then used to record the macroscopic morphological evolution of the anode and cathode, as well as changes in surface pH, while scanning electron microscopy (SEM) and other techniques were applied to examine the microstructural characteristics of dendrites. In addition, an electrochemical workstation was utilized to monitor, in real time, the short-circuit failure time and current of solder alloy electrode pairs. Based on these observations, the electrochemical migration resistance of SAC305–*x*Ni solder alloys in a simulated dew condensation environment was systematically evaluated and discussed.

## 2 Experimental

### 2.1 Materials

Commercially pure Sn (99.99%), Ag (99.99%), Cu (99.99%), and Ni (99.6%) were used as raw materials to prepare four SnAgCuNi alloys with compositions of SAC305–0.05Ni, SAC305–0.1Ni, SAC305–0.25Ni, and SAC305–0.5Ni. The weighed raw materials were placed into a vacuum melting furnace, evacuated, and then purged with argon gas to clean the furnace chamber. After melting, once the alloy surface became fully liquid, the molten metal was poured into a mold and air-cooled to room temperature. For parallel control experiments, commercially available Sn96.5Ag3.0Cu0.5 (SAC305) solder alloy was used. The detailed compositions of the solder alloys are listed in Table 1.

**Table 1:** Chemical composition of SAC305 solder alloy (wt%)

Alloy	Sn	Ag	Cu	Pb	Sb	Ni	Bi	Fe	In
SAC305	Bal.	2.968	0.523	0.008	0.011	—	0.002	0.004	0.003
SAC305–0.05Ni	Bal.	2.968	0.523	0.008	0.011	0.050	0.002	0.004	0.003
SAC305–0.10Ni	Bal.	2.968	0.523	0.008	0.011	0.100	0.002	0.004	0.003
SAC305–0.25Ni	Bal.	2.968	0.523	0.008	0.011	0.250	0.002	0.004	0.003
SAC305–0.50Ni	Bal.	2.968	0.523	0.008	0.011	0.500	0.002	0.004	0.003

**Fig. 1:**
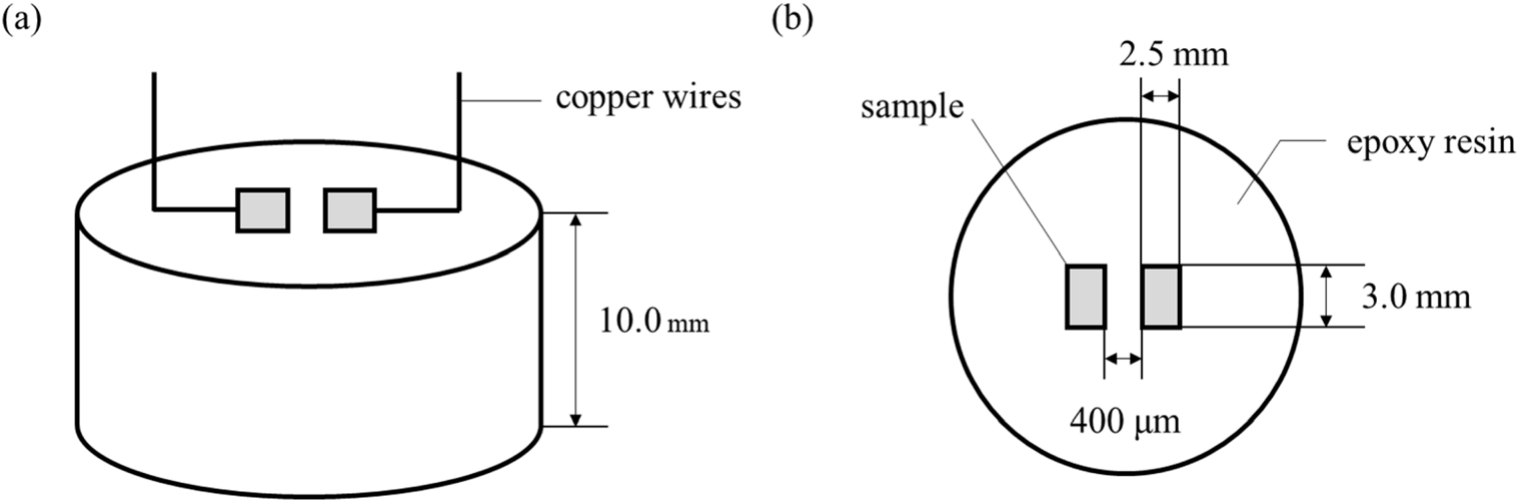
Schematic diagram of electrochemical migration specimen preparation: (a) specimen structure; (b) schematic of the test surface.

The prepared alloys were cut into block specimens with dimensions of 2.5 × 3.0 × 10.0 mm. Each pair of specimens was sealed with epoxy resin, leaving the upper and lower surfaces exposed and separated laterally by a 400 μm gap. After curing at rest, one surface was connected to a copper wire by soldering and the joint was coated with silicone sealant. The opposite surface was sequentially ground with 120, 240, 400, 1000, and 2000 grit abrasive papers, ultrasonically cleaned in deionized water followed by anhydrous ethanol, and then dried for testing. The specimen structure and the designated test surface are illustrated in Fig. 1.

### 2.2 Water drop test

**Fig. 2:**
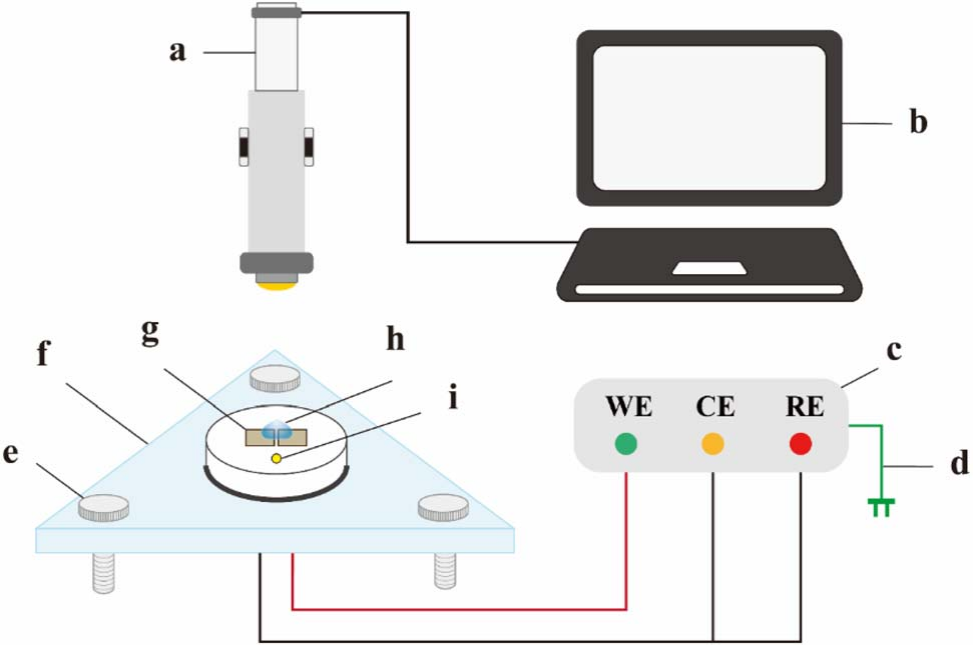
*In situ* water drop test setup: (a) stereomicroscope; (b) display monitor; (c) electrochemical workstation; (d) power supply; (e) leveling knobs; (f) specimen stage; (g) specimen; (h) water droplet; (i) circular spirit level.

An *in situ* observable water drop test was employed to simulate the electrochemical migration (ECM) failure behavior of SAC305–*x*Ni solder alloys under dew condensation conditions, with the overall setup and connection scheme illustrated in Fig. 2. The epoxy-sealed specimens (Fig. 2g) were embedded into the specimen holder (Fig. 2f), and the three leveling knobs of the triangular stage (Fig. 2e) were adjusted to ensure the specimen surface was horizontal, as checked with the circular spirit level (Fig. 2i). A 3 μL droplet of electrolyte solution (Fig. 2h) was dispensed between the two electrodes, and a DC bias of 3 V was applied across them using the potentiostatic mode of an electrochemical workstation (Fig. 2c) to drive the ECM process. The current–time curve was recorded throughout the test in a two-electrode configuration. The electrolyte used was a 30 mM NaCl solution. The surface pH distribution was visualized by adding 3–4 drops of a pH indicator into 50 mL of electrolyte, mixing thoroughly, and comparing the resulting color with a standard color chart. To ensure the accuracy of the water drop ECM tests, each experimental condition was repeated at least three times.

### 2.3 Electrochemical performance testing

To characterize the electrochemical corrosion behavior of SAC305–*x*Ni solder alloys, electrochemical tests were conducted using a VersaSTAT 3F electrochemical workstation in a conventional three-electrode configuration, with the solder alloy specimen (10 mm × 10 mm × 3 mm) as the working electrode, a platinum plate as the counter electrode, and a saturated calomel electrode (SCE) as the reference electrode. Prior to testing, the specimens were ground with 2000 grit abrasive paper, rinsed sequentially with deionized water and anhydrous ethanol, and then dried with compressed air. The potentiodynamic polarization tests were performed at a scan rate of 0.1667 mV s^−1^, while the EIS measurements were carried out with an AC perturbation amplitude of ±10 mV over the frequency range of 0.01 Hz to 100 kHz. The impedance spectra were fitted and analyzed using Zsimpwin software. All electrochemical tests were conducted at 25 °C, and each test was repeated at least three times to ensure the accuracy and reproducibility of the results.

### 2.4 Surface morphology

To systematically investigate the failure mechanisms and corrosion characteristics of SAC305–*x*Ni solder alloys during electrochemical migration, multiple microscopic techniques were employed for morphological and compositional characterization.

First, a KEYENCE VK-X250 3D laser confocal microscope was used to examine the surface corrosion morphology and dendrite growth of SAC305–*x*Ni specimens after ECM failure.

In addition, a ZEISS G300 scanning electron microscope (SEM) was employed to observe and distinguish the failure morphology and dendritic features of SAC305–*x*Ni after ECM. An X-ray energy-dispersive spectroscopy (EDS) system was used to analyze the elemental composition of the dendrites and the corrosion products on both the anode and cathode.

## 3 Results and discussion

**Fig. 3:**
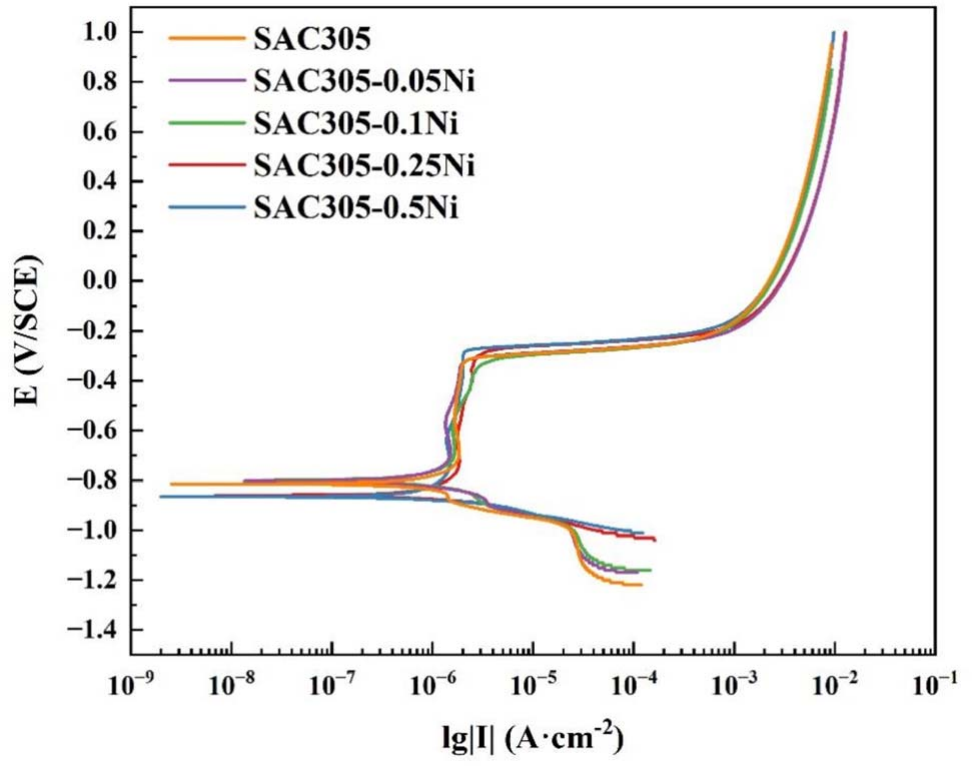
Potentiodynamic polarization curves of SAC305–*x*Ni solder alloys in 30 mM NaCl solution.

The corrosion resistance of the SAC305–*x*Ni alloys were firstly measured by electrochemical tests. Fig. 3 presents the polarization response of SAC305–*x*Ni solder alloys in 30 mM NaCl solution. The polarization curves show obvious active, passive and transpassive behavior due to the formation of the passivation film. The electrochemical characteristics, including corrosion potential (*E*_corr_) and corrosion current density (*i*_corr_), were determined through Tafel extrapolation of the anodic and cathodic branches, with the corresponding quantitative values summarized in Table 2.

**Table 2:** Electrochemical parameters of SAC305–*x*Ni solder alloys obtained by fitting the potentiodynamic polarization curves in Fig. 3

Samples	*E*_corr_ (V/SCE)	*i*_corr_ (μA cm^−2^)
SAC305	−0.80339	3.0360
SAC305–0.05Ni	−0.80039	2.0313
SAC305–0.1Ni	−0.80030	1.5985
SAC305–0.25Ni	−0.86489	2.2057
SAC305–0.5Ni	−0.85982	3.0362

In 30 mM NaCl solution, the *E*_corr_ of the SAC305 solder alloy is −803.39 mV (*vs.* SCE), with *i*_corr_ of 3.036 μA cm^−2^. When a small amount of Ni (0.05–0.1 wt%) is introduced, the *E*_corr_ slightly shifts in the positive direction (−800.39 to −800.30 mV), while the *i*_corr_ decreases significantly to 2.0313 μA cm^−2^ and 1.5985 μA cm^−2^, respectively. This indicates that a low Ni content can effectively reduce the corrosion rate and improve the corrosion resistance of the solder alloy. However, as the Ni content is further increased to 0.25 wt% and 0.5 wt%, the *E*_corr_ values shift negatively (−864.89 and −859.82 mV), accompanied by an increase in *i*_corr_ to 2.2057 μA cm^−2^ and 3.0362 μA cm^−2^. These results suggest that Ni addition initially enhances the corrosion resistance of SAC305, with the best performance observed at 0.1 wt% Ni, but excessive Ni addition deteriorates the protective effect.

**Fig. 4:**
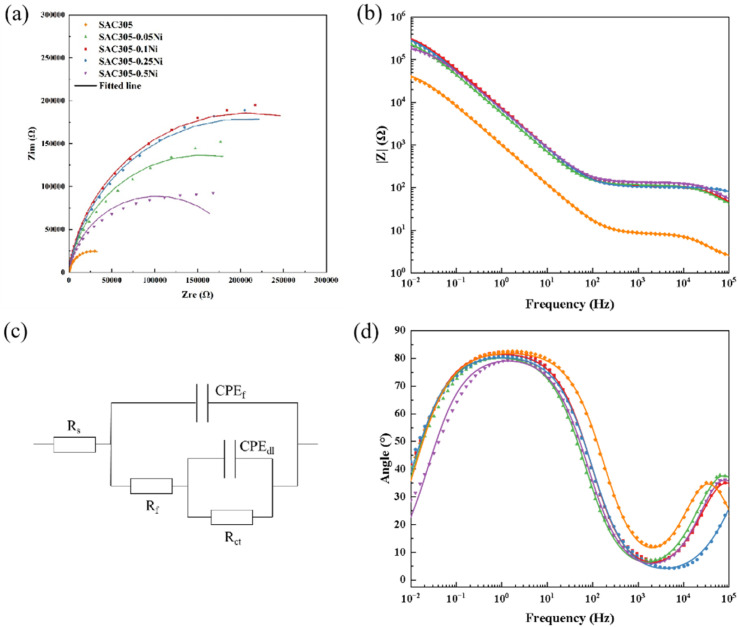
EIS results of SAC305–*x*Ni solder alloys in 30 mM NaCl solution: (a) Nyquist plots; (b) Bode plots; (c) equivalent circuit model used for data fitting; and (d) Bode phase angle plots.

The EIS results of SAC305–*x*Ni solder alloys with different Ni contents are presented in Fig. 4. As shown in the Bode plots (Fig. 4b and d), two distinct time constants can be observed in the impedance spectra. The high-frequency response is associated with the surface corrosion products, corresponding to the solution resistance (*R*_s_), film capacitance (CPE_f_), and film resistance (*R*_f_). In contrast, the low-frequency region reflects the charge transfer process at the alloy/electrolyte interface, which is represented by the double-layer capacitance (CPE_dl_) and the charge transfer resistance (*R*_ct_). Accordingly, the equivalent circuit model in Fig. 4c was employed to fit the experimental spectra. Since the Nyquist plots (Fig. 4a) do not show perfect semicircles and the maximum phase angle in the Bode plots is below 90°, deviations from ideal capacitive behavior are indicated. These features can be attributed to surface roughness, non-uniform current distribution, porous film effects, and frequency dispersion. Therefore, a constant phase element (CPE) was introduced into the fitting circuit to replace the ideal capacitor. As reported in previous studies,^23^ the parameters of the CPE include the admittance (*Y*_o_), the imaginary unit (j), the angular frequency (*ω*), and the dispersion index (*n*, 0 < *n* < 1), which characterizes the degree of deviation from ideal capacitive behavior.

The EIS fitting results of SAC305–*x*Ni solder alloys based on the equivalent circuit (Fig. 4c) are summarized in Table 3. From the perspective of charge transfer resistance (*R*_ct_), the base SAC305 alloy exhibits a relatively low value of 5.60 × 10^4^ Ω cm^2^. With the addition of Ni, the *R*_ct_ increases significantly, reaching 4.12 × 10^5^ Ω cm^2^ at 0.05 wt% Ni and peaking at 4.19 × 10^5^ Ω cm^2^ when the Ni content is 0.1 wt%. This pronounced increase indicates that a small amount of Ni addition can effectively inhibit interfacial charge transfer and enhance the corrosion resistance of the solder alloy. However, as the Ni content is further increased to 0.25 wt% and 0.5 wt%, the *R*_ct_ decreases to 3.13 × 10^5^ and 2.68 × 10^5^ Ω cm^2^, respectively. This decline suggests that excessive Ni leads to deterioration in the stability of the protective film and a reduction in overall corrosion resistance.

**Table 3:** EIS fitting results of SAC305–*x*Ni alloys

SAC305–*x*Ni	*R*_s_ (Ω cm^2^)	Passive film			Electric double layer		
		*Y*_o,f_ (F cm^−2^ s*^n^*^−1^)	*n*_f_	*R*_f_ (Ω cm^2^)	*Y*_o,dl_ (F cm^−2^ s^*n*−1^)	*n*_dl_	*R*_ct_ (Ω cm^2^)
*x* = 0	23	2.960 × 10^−7^	0.94	60	1.81 × 10^−4^	0.92	5.60 × 10^4^
*x* = 0.05	28.6	1.100 × 10^−7^	0.81	105	2.76 × 10^−5^	0.91	4.12 × 10^5^
*x* = 0.10	20.7	1.732 × 10^−7^	0.90	87	2.51 × 10^−5^	0.93	4.19 × 10^5^
*x* = 0.25	29.6	2.110 × 10^−7^	0.89	98	3.37 × 10^−5^	0.91	3.13 × 10^5^
*x* = 0.50	20.4	1.931 × 10^−7^	0.93	103	2.71 × 10^−5^	0.91	2.68 × 10^5^

These electrochemical results are consistent with previous finding.^13^ At this level, Ni promotes the formation of a dense and stable passivation film, effectively lowering the corrosion current density and significantly increasing the charge transfer resistance. Moreover, surface analysis revealed that the corrosion products at 0.05–0.1 wt% Ni are primarily composed of compact Sn(iv)-based oxides, which reduce chloride ion penetration and limit pit depth (15.7 μm compared to 21.4 μm for SAC305). In contrast, higher Ni contents (0.25–0.5 wt%) favor the formation of porous passivation films containing Sn(OH)_4_ and Sn–Cl complexes, accompanied by deeper pits (up to 40.9 μm) and enhanced intergranular corrosion. These observations clarify that a small Ni addition stabilizes the corrosion product layer and enhances protective performance, whereas excessive Ni leads to galvanic coupling and deterioration of film integrity.

**Fig. 5:**
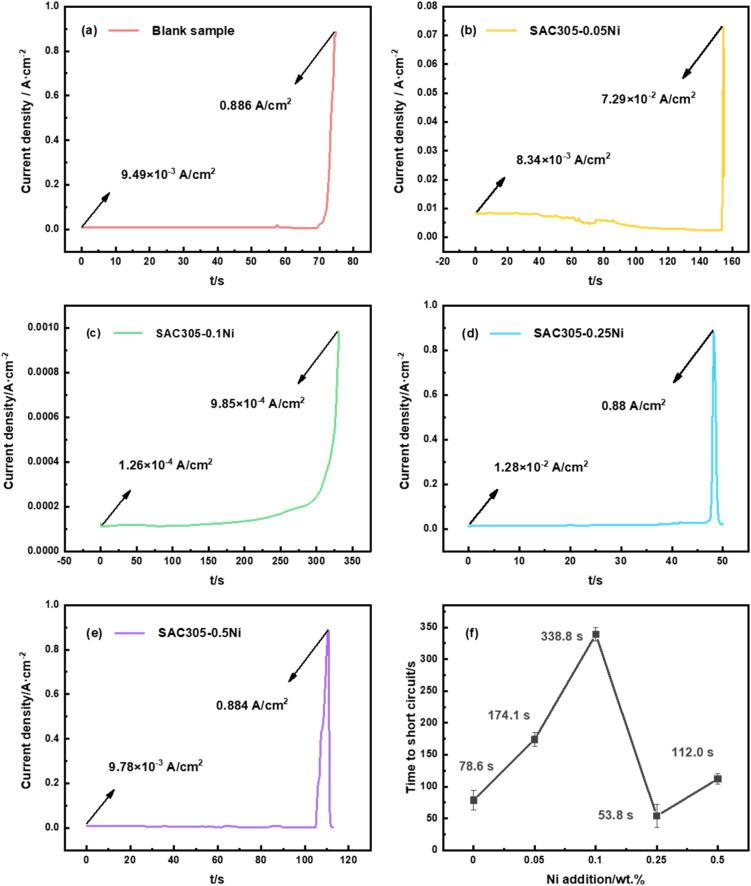
Electrochemical migration current–time curves of (a–e) SAC305–*x*Ni solder alloys (*x* = 0, 0.05, 0.10, 0.25, 0.50 wt%) under a 30 mM NaCl water drop test at 3 V bias; (f) short-circuit time as a function of Ni addition in SAC305–*x*Ni solder alloys.

Then, a DC bias of 3 V was applied to the solder alloys using an electrochemical workstation, and the current–time curves generated between the electrodes during electrochemical migration of SAC305–*x*Ni alloys were recorded in real time, as shown in Fig. 5. When dendrites grow from the cathode toward the anode, the circuit experiences a sudden surge in current density due to short-circuit failure. As shown in Fig. 5a, under a 30 mM NaCl droplet, the SAC305 alloy exhibited an initial current density of approximately 9.49 × 10^−3^ A cm^−2^ and a peak current density at failure of 0.886 A cm^−2^. In comparison, when the Ni content was 0.05–0.10 wt%, both the initial current density and the peak failure current density were significantly reduced, reaching 1.26 × 10^−3^ A cm^−2^ and 9.85 × 10^−4^ A cm^−2^, respectively. However, at Ni contents of 0.25–0.50 wt%, the initial and peak failure current densities increased again. Fig. 5f shows the variation in short-circuit time with Ni content for SAC305–*x*Ni solder alloys. Compared with SAC305 (78.6 s), SAC305–0.05Ni (174.1 s) and SAC305–0.10Ni (388.8 s) exhibited markedly prolonged failure times, whereas 0.25 wt% Ni accelerated ECM, reducing the short-circuit time to 53.8 s. A Ni content of 0.50 wt% also showed a certain delaying effect.

**Fig. 6:**
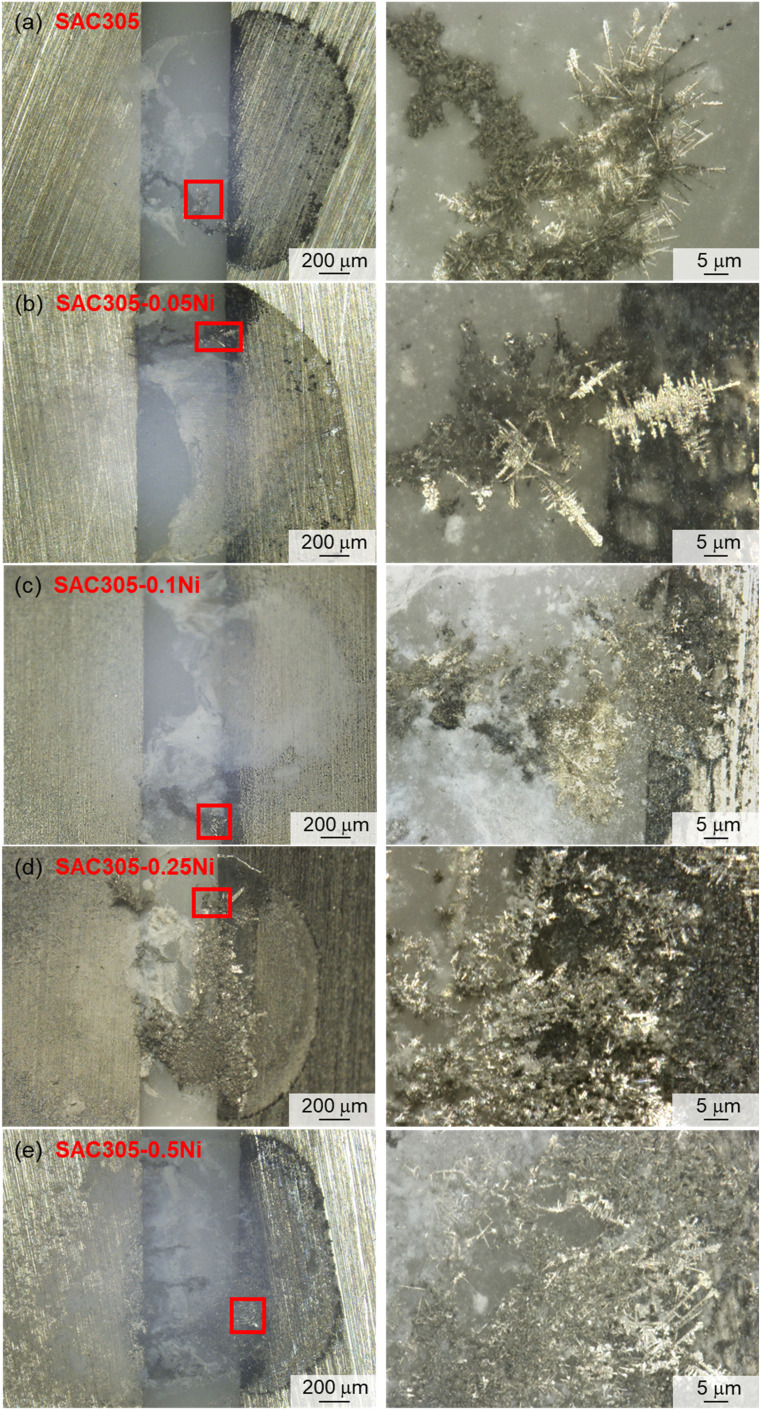
3D laser confocal microscopy images of dendrites in SAC305–*x*Ni solder alloys: (a–e) correspond to *x* = 0, 0.05, 0.10, 0.25, and 0.50 wt%.

**Fig. 7:**
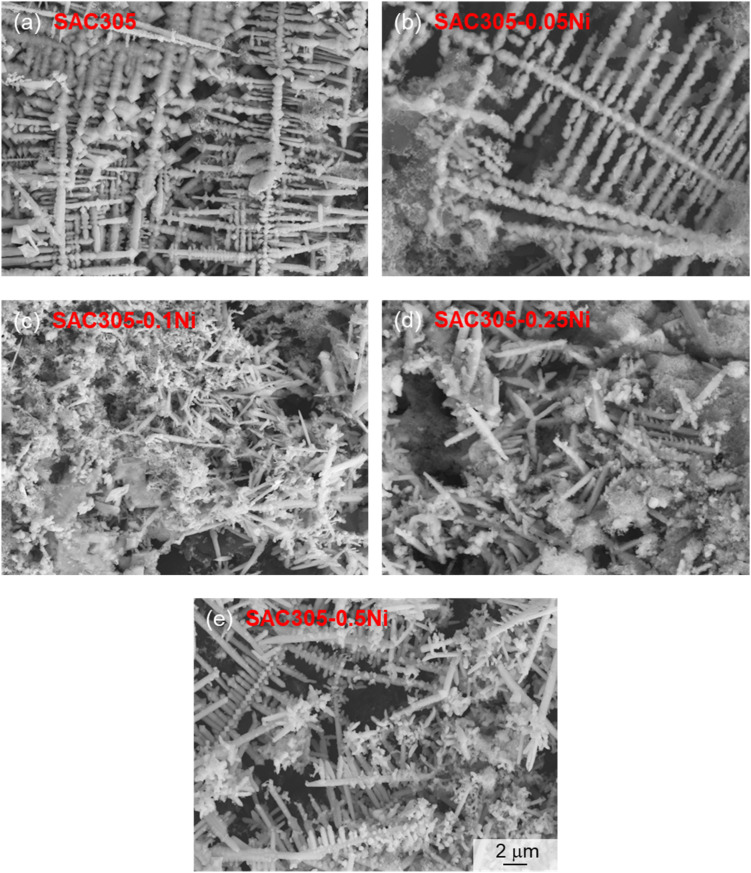
SEM images of dendrites in SAC305–*x*Ni solder alloys: (a–e) correspond to *x* = 0, 0.05, 0.10, 0.25, and 0.50 wt%.

A 3D laser confocal microscope and the SEM were used to examine the electrochemical migration characteristics of SAC305–*x*Ni solder alloys and the micro-morphology of dendrites formed under a 3 V bias, as shown in Fig. 6 and 7. In Fig. 6, the left side corresponds to the cathode and the right side to the anode. Under the 30 mM NaCl water drop test, SAC305 produced only a small amount of white corrosion products on the anode side, which were loosely distributed and sparsely connected to the dendritic growth region. When the Ni content was 0.05–0.10 wt%, the corrosion products near the anode edge became more abundant and densely packed, indicating that Ni addition promotes localized deposition in the early stages of ECM. With further increases in Ni content, the white products progressively accumulated between the two electrodes, forming continuous deposits that bridged toward the cathode edge, suggesting a shift in the migration path and deposition zone.

As shown in Fig. 7a, the dendrites formed on SAC305 were thin, elongated, and highly branched, with numerous secondary nucleation sites along the main stem, indicating an unstable and rapidly propagating growth front. With 0.10 wt% Ni addition, the dendrites became shorter and finer, and branching was significantly reduced, suggesting suppression of long-range ionic transport and localized growth kinetics, which corresponds well to the delayed current rise and reduced peak current density observed in Fig. 5. However, when the Ni content was further increased to 0.25–0.50 wt%, the dendrites became thicker and more robust, with fewer but longer side branches growing from the main stem, reflecting a recovery of growth kinetics and a possible reduction in the passivation effect. These morphological changes clearly demonstrate that Ni content plays a dual role, initially inhibiting and then enhancing ECM growth, depending on the concentration range.

**Fig. 8:**
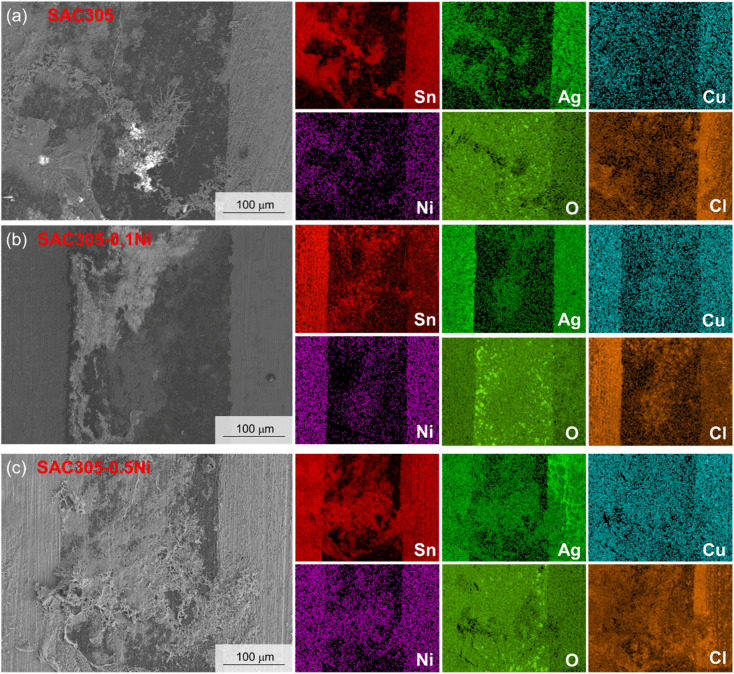
EDS area scan results of dendrites in SAC305–*x*Ni solder alloys: (a–c) correspond to *x* = 0, 0.10, and 0.50 wt%.

To investigate the metallic elements involved in migration during the ECM process of SAC305–*x*Ni and to clarify the composition of the dendritic products, EDS area scanning was performed on the failed surfaces, and the results are shown in Fig. 8. In the 30 mM NaCl water drop test, a substantial migration of Sn and Ag occurred in SAC305, while Cu migration was minimal or almost absent. In SAC305–0.10Ni, Sn, Ag, Ni, and Cu all underwent dissolution and migration. As shown in Fig. 8b, Cl was also found to accumulate between the two electrodes, suggesting that the products may contain small amounts of metal chlorides. For the SAC305–0.50Ni alloy, Sn, Ag, and Ni were enriched in the inter-electrode products, whereas Cu exhibited a relatively uniform distribution.

These results indicate that Ni addition alters the migration behavior of multiple elements in the alloy. At low Ni contents (*e.g.*, 0.10 wt%), Ni may participate in local electrochemical reactions, facilitating co-dissolution of Cu with Sn and Ag, potentially through modification of the protective intermetallic layers. The presence of Cl^−^ between the electrodes further suggests the formation of soluble or sparingly soluble metal chlorides, which could facilitate ionic transport and dendrite growth. From an electrochemical perspective, this observation is consistent with polarization and EIS results, where 0.05–0.10 wt% Ni alloys exhibit slightly nobler corrosion potentials, reduced corrosion current densities, and significantly higher charge transfer resistances compared with the base SAC305 alloy, confirming that limited Ni addition improves passivation and hinders interfacial charge transfer. At higher Ni contents (*e.g.*, 0.50 wt%), the accumulation of Sn, Ag, and Ni in the inter-electrode deposits, combined with the more uniform Cu distribution, implies a shift in the dominant migration pathway, where Ni-rich phases may act as preferential dissolution sites, while Cu remains largely immobilized in the bulk microstructure. This compositional shift correlates with the more negative corrosion potentials and reduced *R*_ct_ values observed in electrochemical tests, suggesting that excessive Ni promotes galvanic coupling and destabilizes the passive film, thereby reducing overall corrosion resistance during ECM.

**Fig. 9:**
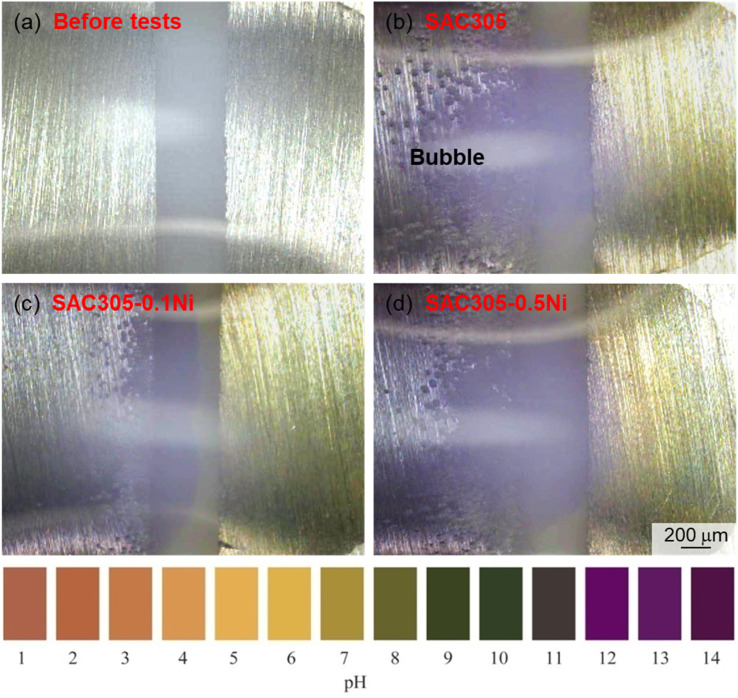
Surface pH distribution of SAC305–*x*Ni alloys during electrochemical migration: (a) before test for reference; (b–d) after test for SAC305, SAC305–0.10Ni, and SAC305–0.50Ni, respectively.

The surface pH of SAC305–*x*Ni during electrochemical migration was visualized using a diluted pH indicator solution (30 mL commercial pH indicator + 100 mL deionized water), and the results are shown in Fig. 9. During the ECM process, the cathode initially undergoes oxygen and water reduction reactions,^24–26^ as follows:

1 O_2_ + 2H_2_O + 4e^−^ → 4OH^−^

2 2H_2_O + 2e^−^ → H_2_ + 2OH^−^

These reactions produce hydroxide ions (OH^−^), leading to a local increase in pH near the cathode surface. Due to the generation of a large amount of OH^−^ ions at the cathode, the cathodic side of the droplet appears purple, indicating a gradual increase in alkalinity. The OH^−^ ions then migrate toward the anode under the combined influence of the concentration gradient and the electric field. In addition, the vigorous hydrogen evolution reaction at the cathode produces hydrogen gas, which strongly agitates the solution within the droplet, further facilitating the migration of OH^−^. Moreover, the relatively small ionic radius of hydroxide ions allows for a faster migration rate. Consequently, cations and anions combine and react in the vicinity of the anode.

**Fig. 10:**
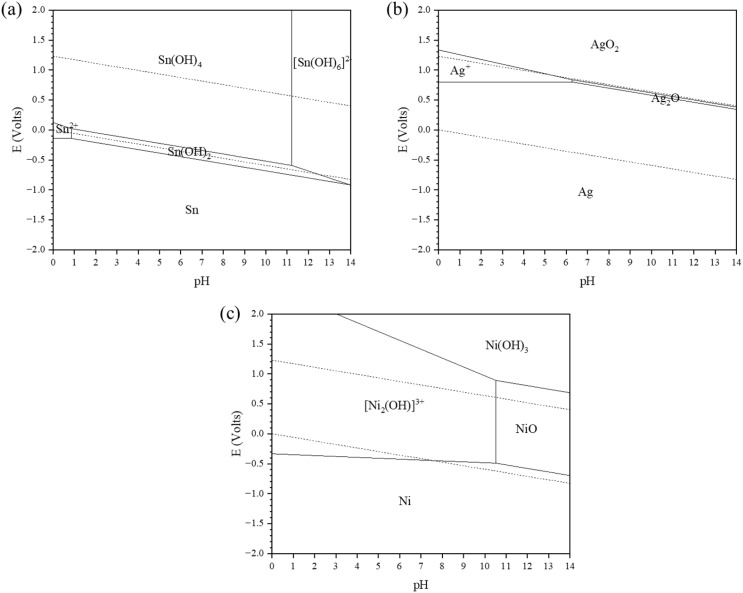
*E*–pH (Pourbaix) diagrams of (a) Sn, (b) Ag, and (c) Ni in the H_2_O system at 25 °C.

At the same time, metal oxidation occurs at the anode, producing dissolved metal cations. According to the compositional analysis results, Sn, Ag, Cu, and Ni all undergo migration in SAC305–*x*Ni, with Sn, Ag, and Ni participating predominantly in the ECM process. This may be attributed to the relatively high stability of the intermetallic compound (Ni,Cu)_6_Sn_5_,^27^ which can partially reduce the electrochemical migration susceptibility of Cu. Insights from Pourbaix diagrams (Fig. 10) further support these behaviors: Sn is prone to dissolve as Sn^2+^ under acidic conditions and can be oxidized to Sn^4+^ at higher potentials, while in neutral to alkaline environments it tends to form insoluble hydroxides such as Sn(OH)_2_ and Sn(OH)_4_, indicating a passivation tendency. Ag exhibits a wide stability region in its metallic state, reflecting its inherent resistance to corrosion, but at more oxidizing potentials Ag^+^ or Ag_2_O can form, which still enables migration under ECM conditions. Ni is unstable in acidic solutions where it dissolves as Ni^2+^, whereas in neutral to alkaline environments it forms protective oxides/hydroxides (NiO, Ni(OH)_2_), which may further oxidize to NiOOH at high potentials. Therefore, the following anodic reactions are likely to occur near the anode:

3 Sn → Sn^2+^ + 2e^−^

4 Ag → Ag^+^ + e^−^

5 Ni → Ni^2+^ + 2e^−^

For Sn, Sn(ii) may be oxidized to Sn(iv) *via* reaction (6), and subsequently undergo hydrolysis and precipitation near the anode. Given that *K*_sp_ for Sn(OH)_4_ is 10^−57^ and *K*_sp_ for Sn(OH)_2_ is 10^−27^,^28^ precipitation is expected to occur at the anode, as shown in reactions (7) and (8).

6 Sn^2+^ → Sn^4+^ + 2e^−^

7 Sn^2+^ + 2OH^−^ → Sn(OH)_2_

8 Sn^4+^ + 4OH^−^ → Sn(OH)_4_

Since Sn(OH)_4_ is an amphoteric hydroxide with acidic properties, it exhibits good solubility in alkaline environments and can further react with hydroxide ions to form [Sn(OH)_6_]^2−^. Consequently, the resulting metal ions and Sn-containing species migrate together toward the cathode, where they are eventually reduced and deposited, leading to dendrite formation.^29^

9 Sn(OH)_4_ + 2OH^−^ → [Sn(OH)_6_]^2−^

10 [Sn(OH)_6_]^2−^ + 4e^−^ → Sn + 6OH^−^

11 Sn^2+^ + 2e^−^ → Sn

12 Sn^4+^ + 4e^−^ → Sn

For Ag, the oxidation reaction generates Ag^+^ ions, which transiently combine with OH^−^ to form AgOH. However, AgOH is unstable at room temperature and readily decomposes into Ag_2_O and water, as shown in reaction (13). Silver oxide exhibits good solubility in alkaline environments; thus, the resulting metal ions and Ag-containing species migrate together toward the cathode, where they are ultimately reduced and deposited, leading to dendrite formation, as shown in reaction (15).

13 2AgOH → Ag_2_O + H_2_O

14 Ag_2_O + H_2_O + 2e^−^ → 2Ag + 2OH^−^

15 Ag^+^ + e^−^ → Ag

For Ni, Ni(ii) combines with OH^−^ near the anode to form Ni(OH)_2_, while a portion of the hydroxide is further oxidized to NiOOH and related species, as shown in reactions (16) and (17). NiOOH behaves similarly to basic salts and undergoes reduction to Ni(OH)_2_, which then releases Ni^2+^ that migrates toward the cathode. These Ni-containing species are eventually reduced to metallic Ni, contributing to dendrite growth, as shown in reactions (18) and (19).

16 Ni^2+^ + 2OH^−^ → Ni(OH)_2_

17 Ni(OH)_2_ + OH^−^ → NiOOH + e^−^ + H_2_O

18 NiOOH + H_2_O + e^−^ → Ni(OH)_2_ + OH^−^

19 Ni^2+^ + 2e^−^ → Ni

Previous studies have shown that low concentrations of chloride ions have little effect on the constructed Pourbaix diagram of the Sn–H_2_O system.^30,31^ However, EDS area scan results indicate the presence of small amounts of metal chlorides between the two electrodes, suggesting that a fraction of the metals may dissolve into the droplet through oxidation reactions and subsequently react with Cl^−^, as shown in reactions (20)–(22).

20 Sn^2+^ + 2Cl^−^ + 2H_2_O → SnCl_2_·2H_2_O

21 Ag^+^ + Cl^−^ → AgCl

22 Ni^2+^ + 2Cl^−^ + 6H_2_O → NiCl_2_·6H_2_O

For SAC305, the hydrogen evolution reaction during the water drop test generated a large number of bubbles on the cathodic side, with an instantaneous pH of approximately 13. In SAC305–0.10Ni, the bubbles exhibited a smaller diffusion range, were mostly concentrated along the edges, and the alkalinity was slightly reduced. In contrast, the hydrogen evolution reaction at the cathode of SAC305–0.50Ni was more intense. This may indicate that the addition of a small amount of Ni promotes the stable formation of intermetallic compounds (IMCs), increasing the resistance to anodic metal dissolution and thereby reducing the corrosion rate; consequently, the hydrogen evolution reaction at the cathode is also weakened on a macroscopic level. However, with further increases in Ni content, the excess Ni ions generated through anodic ionization participate in the ECM process. On one hand, the increased ionic concentration in the droplet enhances the conductivity of the electrolyte; on the other hand, the higher concentration of metal ions accelerates their cathodic reduction, leading to rapid dendrite growth and ultimately accelerating the ECM failure of the alloy. From a practical perspective, these findings suggest that low Ni additions (0.05–0.10 wt%) not only optimize electrochemical performance but also improve the long-term reliability of SAC305 solder joints under humid service conditions. This indicates that controlling Ni content provides a feasible approach to enhance the durability of Pb-free solders in harsh environments.

## 4 Conclusions

This work focuses on the ECM failure behavior of SnAgCuNi solder alloys, including SAC305–0.05Ni, SAC305–0.10Ni, SAC305–0.25Ni, and SAC305–0.50Ni. An *in situ* observable water drop test was employed to simulate the ECM failure of SAC305–*x*Ni solder alloys under dew condensation conditions. A stereomicroscope was used to record the macroscopic morphology of the anode and cathode as well as changes in surface pH. Surface analysis techniques were applied to examine the microstructure of dendrites, while an electrochemical workstation was used to record in real time the short-circuit failure time and current between solder alloy electrode pairs. Based on these measurements, the ECM resistance of SAC305–*x*Ni solder alloys in a simulated dew condensation environment was analyzed. The main conclusions are as follows:

(1) In 30 mM NaCl solution, SAC305–0.05Ni and SAC305–0.10Ni exhibited slightly nobler corrosion potentials and markedly reduced corrosion current densities compared with SAC305, while Ni additions of 0.25–0.50 wt% resulted in more negative *E*_corr_ values and higher *i*_corr_. Consistently, EIS analysis showed that 0.05–0.10 wt% Ni significantly increased the charge transfer resistance (*R*_ct_) by nearly one order of magnitude, whereas further Ni addition led to a decline in *R*_ct_, indicating deterioration of corrosion resistance.

(2) In a 30 mM NaCl water drop, SAC305–0.05Ni and SAC305–0.10Ni exhibited significantly reduced initial current density and peak current density at failure compared with SAC305, whereas Ni additions of 0.25–0.50 wt% led to increased initial and peak failure current densities. Moreover, SAC305–0.05Ni and SAC305–0.10Ni showed a marked extension of failure time, reaching up to 388.8 s, while 0.25 wt% Ni accelerated ECM in the solder alloy.

(3) In a 30 mM NaCl water drop, dendrites produced by SAC305 during ECM were thin and long, with numerous secondary nucleation sites along the main stem. With increasing Ni content, dendrites in SAC305–0.10Ni became shorter and finer. However, at Ni contents of 0.25–0.50 wt%, dendrites became thick again, with longer branches growing from the main stem. For SAC305–*x*Ni alloys, the primary migrating metallic elements were Sn, Ag, and Ni, while Cu migration was minimal. White anodic products were also observed, which may consist of metal oxides or chlorides.

(4) The addition of 0.05–0.10 wt% Ni helps reduce the ECM reaction rate, lower the ion concentration in the droplet, and mitigate corrosion in lead-free solder alloys, thereby improving the ECM resistance of SAC305. In contrast, adding 0.25 wt% Ni degrades ECM resistance.

## Conflicts of interest

The authors declare that they have no known competing financial interests or personal relationships that could have appeared to influence the work reported in this paper.

## Data Availability

The data of this study are available from the corresponding author upon reasonable request.
